# CD163: a potential biomarker for lupus nephritis activity

**DOI:** 10.1136/lupus-2026-002166

**Published:** 2026-08-10

**Authors:** Erdem Gürel, Suzan Çinar, Özge Hürdogan, Yasemin Özlük, Gamze Kemeç, Ömer Uludağ, Sibel Varelci, Safak Mirioğlu, Yasemin Yalçinkaya, Ahmet Gül, Murat Inanç, Bahar Artim-Esen

**Affiliations:** 1 Division of Rheumatology, Istanbul University Istanbul School of Medicine, Istanbul, Turkey; 2 Department of Immunology, Istanbul University Aziz Sancar Institute of Experimental Medicine, Istanbul, Turkey; 3 Department of Pathology, Istanbul University Istanbul School of Medicine, Istanbul, Turkey; 4 Department of Internal Medicine, Istanbul University Istanbul School of Medicine, Istanbul, Turkey; 5 Division of Nephrology, Istanbul University Istanbul School of Medicine, Istanbul, Turkey

**Keywords:** Systemic Lupus Erythematosus, Lupus Nephritis, Autoimmune Diseases

## Abstract

**Objectives:**

CD163 is a macrophage-associated scavenger receptor shed during inflammatory activation, resulting in measurable soluble CD163 in serum (s) and urine (u). Elevated levels have been reported in inflammatory and immune-mediated conditions, including lupus nephritis (LN). This study aimed to investigate the relationship between soluble CD163 levels and intrarenal CD163 expression, evaluating their association with disease activity and treatment response in LN.

**Methods:**

Forty-five patients with systemic lupus erythematosus (SLE) were included (20 active LN, 10 inactive LN, 15 active extra-renal SLE), together with 20 matched healthy controls. Serum and urine samples were collected at baseline alongside renal biopsy in active LN; 6-month follow-up samples were available for 12 active LN patients. CD163 was measured by ELISA; urinary CD163 was normalised to spot urine creatinine. Renal biopsies from 20 active LN patients were evaluated for CD163+ macrophage density.

**Results:**

uCD163 was higher in active LN (a renal Systemic Lupus Erythematosus Disease Activity Index 2000 (SLEDAI-2K) flare (UPCR ≥0.5 g/g) with biopsy-proven active lesions) than in inactive LN and active extra-renal SLE (both p<0.001), discriminating active from inactive LN (area under the curve (AUC) 0.97) and from extra-renal SLE (AUC 0.89). uCD163 correlated with the National Institutes of Health activity and chronicity indices activity index (ρ=0.55, p=0.016) and with glomerular CD163+ macrophage density (the number of CD163+ macrophages per glomerulus quantified by immunohistochemistry; ρ=0.65, p=0.006), but not with chronicity. Glomerular CD163+ macrophage density also correlated with sCD163 (ρ=0.60, p=0.01) and uCD163 (ρ=0.63, p=0.009). After 6-month induction, uCD163 fell from 7.41 to 1.55 ng/mg (p<0.001).

**Conclusion:**

uCD163 distinguishes active LN from inactive renal and extra-renal disease and tracks treatment response. The association with intrarenal CD163+ macrophages supports its biological relevance as a non-invasive marker of renal inflammation, complementary to histopathology when repeat biopsy is not feasible. These findings support uCD163 as a candidate non-invasive tool for identifying active LN and monitoring treatment response in SLE, warranting validation in larger prospective cohorts.

**WHAT IS ALREADY KNOWN ON THIS TOPIC**

**WHAT THIS STUDY ADDS**

- Urinary soluble CD163 (uCD163) discriminates active lupus nephritis from inactive renal disease and from active extra-renal systemic lupus erythematosus (SLE), and decreases markedly following 6 months of induction therapy.
- uCD163 correlates with intrarenal CD163+ macrophage density and the National Institutes of Health activity index, directly linking a non-invasive urinary biomarker to histopathological evidence of renal inflammation in the same patients.

**HOW THIS STUDY MIGHT AFFECT RESEARCH, PRACTICE OR POLICY**

- uCD163 may serve as a clinically useful non-invasive adjunct for monitoring LN activity and treatment response when repeat biopsy is not feasible and warrants validation in larger, multicentre prospective cohorts before incorporation into routine clinical practice.

## Introduction

 Systemic lupus erythematosus (SLE) is a chronic, multisystemic, inflammatory autoimmune disease characterised by the deposition of immune complexes in tissues, leading to flares and remissions.^1^ Lupus nephritis (LN), which occurs in approximately 50% of patients diagnosed with SLE, remains a major cause of morbidity and mortality. Despite current treatments, many patients progress to chronic kidney insufficiency and end-stage renal disease.^2^

Proteinuria, anti-double-stranded DNA (anti-dsDNA), and serum complement are routinely used to monitor LN activity and treatment response,^3^ but reflect the underlying renal pathogenesis imperfectly, while renal biopsy—the gold standard—is invasive and cannot be repeated.^4^ Non-invasive biomarkers that detect flares early and objectively track treatment response are therefore needed.

CD163 is a class B transmembrane protein that belongs to the scavenger receptor cysteine-rich (SRCR) superfamily and is expressed on the surface of adult monocytes and macrophages.^5^ The essential function of the CD163 receptor is to act as a scavenger for haemoglobin-haptoglobin complexes formed by erythrocyte haemolysis in the intravascular space.^6^ Through CD163-mediated endocytosis, these complexes are phagocytosed by macrophages and are subsequently degraded. This process protects tissues from haemoglobin- and iron-related oxidative stress and damage.^7^

CD163 is predominantly expressed on macrophages.^8^ CD163+ macrophages have been shown to be present in fibrous-crescent and proliferative renal lesions, as well as in acute tubulointerstitial inflammation, in kidney biopsy specimens from patients with LN.^9^ During inflammation, tissue metalloproteinases cleave CD163 from the macrophage surface, releasing it into the circulation as soluble CD163, which is subsequently excreted in the urine.^10^ Previous studies have reported elevated urinary (u) and serum (s) CD163 levels in LN and antineutrophil cytoplasmic antibody-associated vasculitis with renal involvement.^11 12^ Furthermore, the decrease in uCD163 levels following effective treatment in LN patients suggests that uCD163 can be a candidate biomarker to objectively assess treatment response as well as disease flare.^13^

The highlighted association between soluble CD163 levels and disease activity in SLE and LN in the recent studies needs to be validated in different cohorts.^14–17^ Moreover, data demonstrating the correlation between sCD163 and uCD163 levels and intrarenal CD163 expression in LN are scarce.

Therefore, in this longitudinal study, we aimed to investigate the relationship between sCD163, uCD163 and intrarenal CD163 expression in kidney biopsy specimens and to evaluate their association with disease activity and treatment response in LN.

## Methods

### Patients and control groups

This single-centre longitudinal study was conducted at Istanbul University Faculty of Medicine, Rheumatology Division (April 2020–April 2022). Forty-five SLE patients fulfilling SLICC and ACR classification criteria^18 19^ were included: 20 with active LN, 10 with inactive LN and 15 with active extra-renal SLE. Baseline serum and urine samples were collected from all patients; in active LN patients, sampling was concurrent with renal biopsy. Follow-up samples were obtained from 12 active LN patients at 6 months. Disease activity was assessed using SLEDAI-2K;^20^ scores ≥4 defined active disease, and active renal disease required at least one renal SLEDAI-2K criterion (proteinuria, haematuria, pyuria or casts). Patients with active infections were excluded. Active LN was defined as a renal flare (a renal SLEDAI-2K descriptor—proteinuria (UPCR ≥0.5 g/g), glomerular haematuria, pyuria or cellular casts) with a contemporaneous biopsy showing active proliferative (Class III/IV±V) or membranous (Class V) lesions. Inactive LN denoted biopsy-proven LN in sustained renal remission (UPCR <0.5 g/g, inactive sediment, no recent treatment escalation and ≥12 months from the last flare). Active extra-renal SLE required SLEDAI-2K ≥4 from non-renal domains without a renal descriptor.

The control group consisted of healthy volunteers without any known chronic disease or active infections and was matched with the patient group in terms of age and sex.

Demographic, clinical, laboratory and immunological data, along with disease activity scores, immunosuppressive treatments and corticosteroid doses at the time of sampling, were retrieved from the existing database. Proteinuria was assessed using the protein/creatinine ratio from first morning urine, and eGFR was calculated using the Chronic Kidney Disease Epidemiology Collaboration formula.^21^ Renal biopsies were classified according to the International Society of Nephrology/Renal Pathology Society (ISN/RPS) 2003 system by three experienced nephropathologists;^22^ inactive LN biopsies were re-evaluated concurrently with active LN biopsies. Active LN specimens were additionally stained for CD163 expression. Organ damage was assessed using the SLICC/ACR damage index.^19^

Written informed consent was obtained from all patients and healthy controls prior to enrolment. The study was approved by the Istanbul University Faculty of Medicine Clinical Research Ethics Committee (Decision No: 06, dated 05/03/2021; reference: 18.03.2021-138600) and conducted in accordance with the principles of the World Medical Association Declaration of Helsinki.^23^ The study was supported by the Istanbul University Scientific Research Projects Coordination Unit (Project number: TTU-2021-38289). Patients and members of the public were not directly involved in the design, conduct, reporting or dissemination of this research.

### Measurement of serum and urine CD163 levels

Serum and urine samples were collected from patients and healthy volunteers, with baseline samples obtained concurrently with renal biopsy in patients with active LN. Serum samples were centrifuged at 3000 rpm for 10 min and urine samples at 3500 rpm for 3 min to separate supernatants, which were stored at −80 °C until analysis.

Soluble CD163 levels in serum and urine were measured using a commercially available ELISA kit (Quantikine ELISA, Human CD163; R&D Systems, USA) according to the manufacturer’s instructions.

Optical density was measured at 450 nm using a microplate reader within 30 min of stopping the reaction. A standard curve was generated using a four-parameter logistic regression model, and soluble CD163 concentrations were calculated and expressed as ng/mL. To account for variability in urine concentration, urinary CD163 levels were normalised to spot urine creatinine, measured concurrently in all baseline and 6-month samples by the Jaffe method and expressed as nanograms of CD163 per milligram of creatinine.

### Determination of CD163 expression in renal biopsy preparations

Hematoxylin–Eosin (H&E)-stained sections were reviewed by experienced nephropathologists to select suitable tissue blocks for analysis.

Immunohistochemical staining was performed on 3-μm-thick sections using an automated staining platform (Benchmark XT, Ventana Medical Systems, Tucson, AZ, USA). The staining protocol included antigen retrieval with Cell Conditioning Solution 1 (CC1; EDTA, pH 8) for 98 min, followed by incubation with the primary monoclonal anti-CD163 antibody (clone 10D6, Leica Biosystems) at 37 °C for 32 min. Immunoreactivity was visualised using a diaminobenzidine (DAB) chromogen system and counterstained with Hematoxylin.

**Figure 1:**
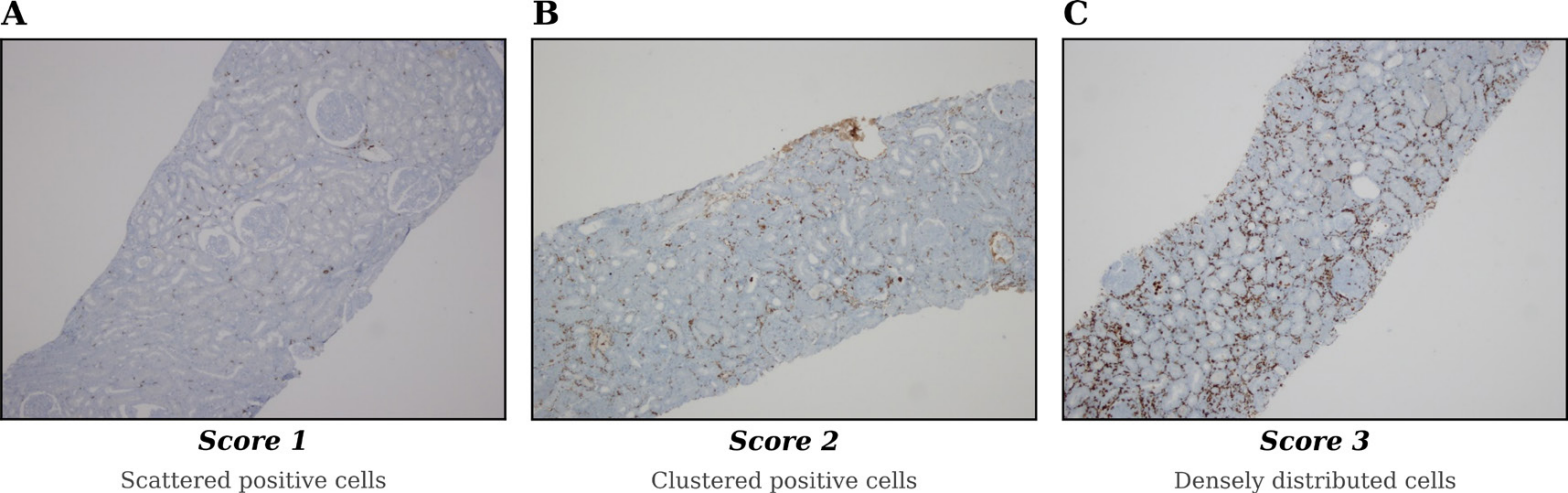
Immunohistochemical scoring of interstitial CD163^+^ macrophages in lupus nephritis biopsies. Representative photomicrographs illustrating the semi-quantitative scoring scheme for tubulointerstitial CD163^+^ infiltrate. (**A**) Score 1 — scattered positive cells. (**B**) Score 2 — clustered positive cells. (**C**) Score 3 — diffusely distributed, densely clustered positive cells. Haematoxylin counterstain. Original magnification ×200–×400.

Cytoplasmic brown staining in inflammatory cells was considered a positive reaction. Interstitial CD163+ cell density was assessed semi-quantitatively using a four-tier scoring system: score 0, no stained cells; score 1, scattered positive cells (figure 1A); score 2, clearly distinguishable and occasionally clustered positive cells (figure 1B); and score 3, diffusely distributed, densely clustered positive cells (figure 1C). The maximum number of CD163^+^ cells within a single capillary lumen was also recorded separately. The number of glomeruli sampled in each biopsy was recorded, and glomerular CD163+ cell density was calculated as the total number of glomerular CD163+ cells divided by the number of glomeruli sampled, expressed as cells per glomerulus. Glomerular and tubulointerstitial CD163+ infiltrates were scored separately.

### Statistical analysis

Statistical analysis was performed using SPSS V.26.0 (IBM Corp., Armonk, NY, USA), and graphs were generated using GraphPad Prism V.9.0 (GraphPad Software, San Diego, CA, USA). The normality of data distribution was assessed using the Shapiro-Wilk and Kolmogorov-Smirnov tests. Continuous variables with normal distribution were expressed as mean±SD, while non-normally distributed variables were presented as median (IQR). Categorical variables were expressed as frequencies and percentages (n, %).

Comparisons between two independent groups were performed using the Student’s t-test or the Mann-Whitney U test, depending on the distribution. For comparisons involving more than two groups, one-way analysis of variance or the Kruskal-Wallis test was applied. Paired comparisons (baseline vs post-treatment) were analysed using the paired t-test or the Wilcoxon signed-rank test. Categorical variables were compared using the χ2 test.

Correlations between biomarkers and clinical/laboratory parameters were evaluated using Pearson’s correlation coefficient (r) for normally distributed data and Spearman’s rank correlation coefficient (rho) for non-normally distributed data. Receiver operating characteristic (ROC) curve analysis was used to evaluate the diagnostic performance of biomarkers, and the area under the curve (AUC) was calculated. The cut-off value for positivity was defined as the mean +2 SD of the healthy control group. All statistical tests were two-tailed, and a p value<0.05 was considered statistically significant. Multiple testing across the correlation panel reported in online supplemental table 1 was controlled by the Benjamini-Hochberg false discovery rate procedure (FDR, α=0.05), and FDR-adjusted q-values are reported alongside p-values. Bootstrap (1000 resamples) 95% CIs are reported for ROC AUCs. Pairwise comparisons of AUCs were performed using the DeLong test, with DeLong 95% CIs reported in table 1.

**Table 1:** Discrimination of active versus inactive lupus nephritis by urinary and serum CD163 and conventional clinical/serological markers (ROC AUC with DeLong 95% CIs; pairwise comparisons against uCD163). Anti-dsDNA was evaluated as positivity at the time of sampling.

Marker	AUC	95% CI	P vs uCD163
UPCR	1.00	1.00 to 1.00	0.25
uCD163	0.97	0.92 to 1.00	reference
Serum C3	0.86	0.73 to 1.00	0.10
Serum C4	0.85	0.71 to 0.99	0.11
Anti-dsDNA	0.73	0.58 to 0.87	0.002
Serum CD163	0.62	0.39 to 0.85	0.006

anti-dsDNA, anti-double-stranded DNA; AUC, area under the curve; ROC, receiver operating characteristic; uCD163, urinary soluble CD163.

## Results

### Baseline characteristics of patients

Forty-five patients with SLE (20 with active LN, 15 with extrarenal active SLE, 10 with inactive LN) and 20 HC participated in the study. The mean age was 35.4±10.6 years in the active LN group and comparable across the other groups. The median disease duration was 35.5 months (range 6–180) in active LN, 69 months in inactive LN and 76 months in extra-renal SLE; the proportion of women was 80%, 70% and 93.3%, respectively, and 75% (n=15) among HCs. Full demographic characteristics of the three SLE groups are presented in online supplemental table 2.

The treatment regimens at the time of sampling are shown in online supplemental table 2. Overall, 84.4% (n=38) were on corticosteroids; the median dose (methylprednisolone) was 28 mg/day in active LN, 8 mg/day in extra-renal SLE and 2 mg/day in inactive LN. Three patients (all extra-renal active SLE) had received rituximab within the preceding 6 months.

### Laboratory and renal biopsy histopathological features in patients with LN

In patients with active LN, the median serum creatinine level was 0.72 (range 0.62–1.43) mg/dL, eGFR was 119.5 (range 43.7–126.6) mL/min/1.73m^2^, CRP was 3.7 (range 0.6–11.2) mg/L, serum C4 was 9 (range 5–22) mg/dL, serum C3 was 65.2±31.8 (range 33–104) mg/dL and mean erythrocyte sedimentation rate was 50.8±30.3 mm/h. Baseline laboratory parameters across the three SLE groups are summarised in online supplemental table 2, and detailed laboratory values for the active and inactive LN groups are shown in online supplemental table 3.

Renal biopsies of patients with LN were evaluated according to the ISN/RPS 2003 classification system. Proliferative nephritis (Class III or IV±V) was present in 17 patients (85%) in the active LN group and 8 patients (80%) in the inactive LN group, whereas pure membranous nephritis (Class V) was seen in three patients (15%) in the active LN group and one patient (10%) in the inactive LN group. The average percentage of sclerotic glomeruli was low in both groups (10.8±7.2 and 7.9±7.5, respectively). The mean National Institutes of Health (NIH) activity score was 7.47±4.07 in the active LN group and 7.80±5.94 in the inactive LN group; the corresponding mean chronicity scores were 0.95±1.22 and 0.80±0.92. Histopathological features are summarised in online supplemental table 3.

### Serum and urine soluble CD163 levels in groups

The positivity threshold was defined as >2 SD above the mean of the HC group. Mean uCD163 levels were significantly higher in patients with active LN compared with healthy controls (8.97±6.39 vs. 1.53±0.40 ng/mg, p<0.001). Similarly, the uCD163 positivity rate was significantly higher in the active LN group (85% vs 5%, p<0.001).

Patients with active LN exhibited significantly higher mean uCD163 levels compared with the inactive LN group (8.97±6.39 vs. 1.45±0.50 ng/mg, p<0.001) and the active extra-renal SLE group (8.97±6.39 vs. 2.19±1.01 ng/mg, p<0.001). The uCD163 positivity rate followed a similar pattern, being significantly higher in active LN (85%) compared with inactive LN (10%, p<0.001) and active extra-renal SLE (47%, p=0.01).

**Figure 2:**
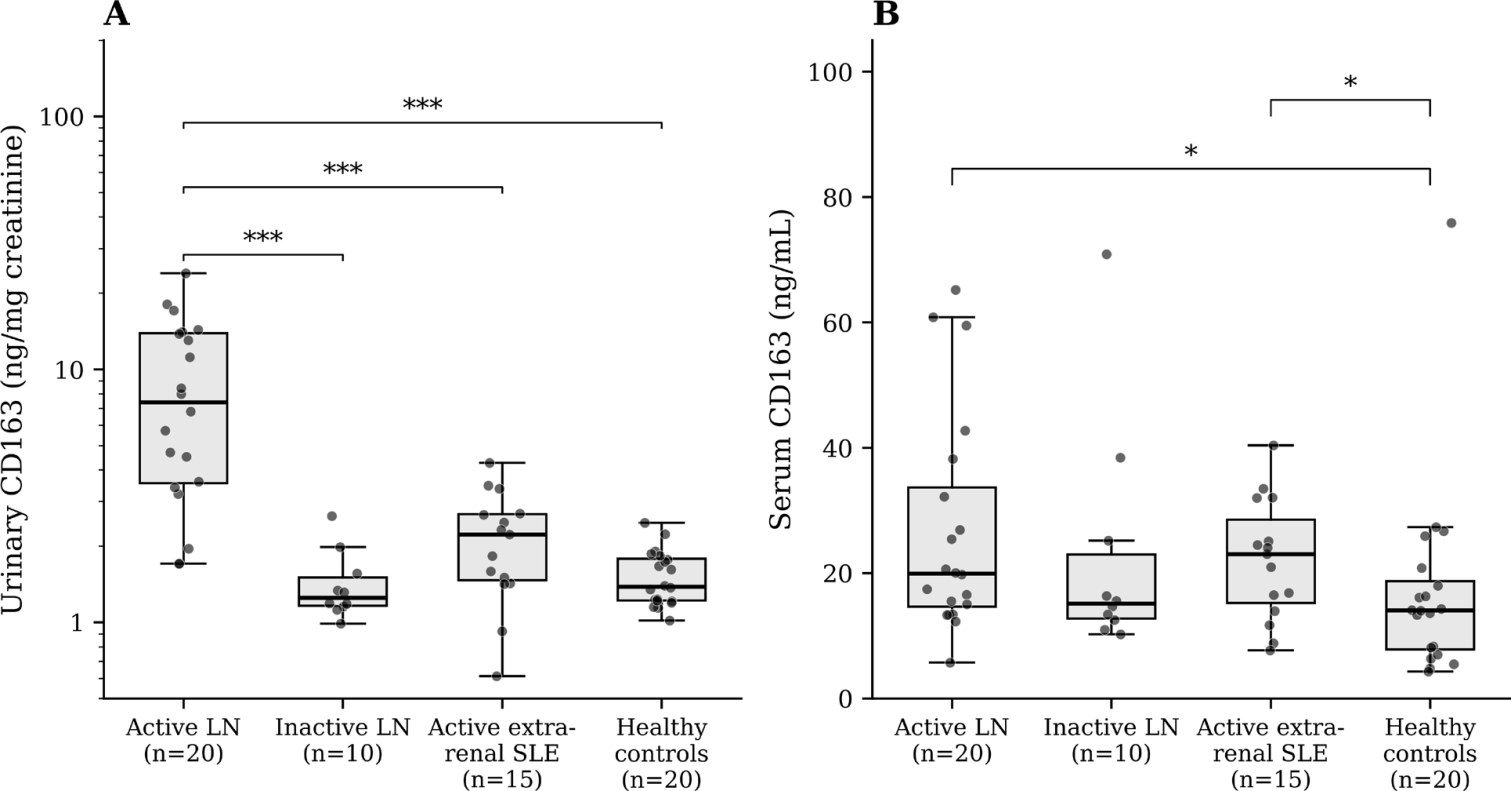
Comparison of urinary and serum CD163 levels across study groups. (**A**) Urinary CD163 normalised to spot urine creatinine (log10 scale). (**B**) Serum CD163. Boxes show median and IQR; whiskers show 1.5× IQR; individual points are overlaid. *n* values are indicated under each group. Group comparisons by Mann–Whitney U test: *p<0.05; ***p<0.001. LN, lupus nephritis; SLE, systemic lupus erythematosus.

Regarding serum levels, the sCD163 positivity rate was higher in the active LN group compared with HC (75% vs 40%, p=0.05). However, no statistically significant difference was found in mean sCD163 levels between the active LN group (26.7±17.6 ng/mL) and healthy controls (17.0±15.6 ng/mL) (p=0.4). Comparisons of sCD163 and uCD163 levels and positivity rates across study groups are presented in figure 2; group-wise uCD163 positivity rates with corresponding Fisher exact comparisons against active LN are tabulated in online supplemental table 4.

Median uCD163 was 7.41 (IQR 3.56–13.90) ng/mg in active LN, compared with 1.25 (1.16–1.51) in inactive LN (p<0.001), 2.23 (1.47–2.68) in active extra-renal SLE (p<0.001) and 1.38 (1.22–1.79) in healthy controls (p<0.001). Full ROC analyses for these comparisons are presented in table 1 and online supplemental figure 1.

### Comparison of serum and urine samples before and after treatment in patients with active LN

**Figure 3:**
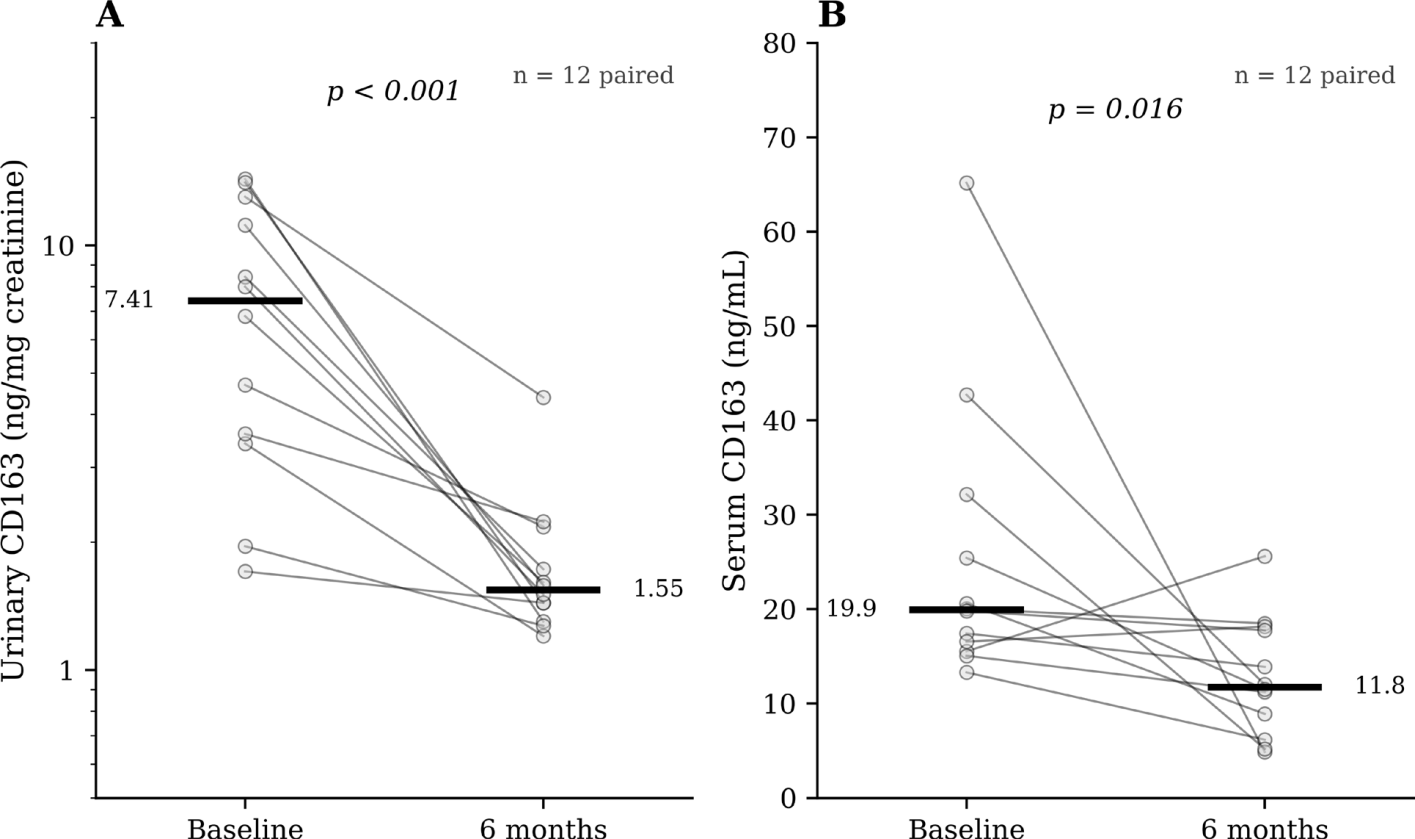
Longitudinal change in soluble CD163 levels in active lupus nephritis (paired baseline and 6-month samples, n=12). (**A**) Urinary CD163 (creatinine-normalised, log10 scale). (**B**) Serum CD163. Each line represents a single patient; horizontal bars indicate group medians. P-values from Wilcoxon signed-rank test.

Follow-up samples were available for 12 of the 20 patients with active LN at the sixth month of induction therapy. A comparison of baseline (pre-treatment) and post-treatment levels revealed a significant reduction in both sCD163 (mean 25.3±15.1 to 12.8±6.3 ng/mL; median 19.9 to 11.8 ng/mL, p=0.016) and uCD163 (mean 7.60±4.67 to 1.82±0.87 ng/mg; median 7.41 to 1.55 ng/mg, p<0.001) levels (figure 3). In paired analysis, uCD163 fell into the range observed in inactive LN at baseline (n=12).

### CD163+ macrophage infiltration in active LN renal biopsies

Immunohistochemical staining for CD163 was performed on renal biopsy specimens from all 20 patients with active LN. CD163+ macrophages were detected in both glomerular and tubulointerstitial compartments.

CD163 interstitial inflammation scores were as follows: score 0 in 0 patients, score 1 in seven patients (35%), score 2 in four patients (20%) and score 3 in nine patients (45%) (representative images shown in figure 1A–C). The mean interstitial inflammation score was 2.1±0.8.

The principal and most novel finding of this study is the demonstration, within the same patients, that intrarenal CD163+ macrophage density is mirrored by circulating and urinary CD163, as summarised in table 2. Within glomeruli, CD163+ macrophages were predominantly localised to mesangial areas and capillary lumens. The median total number of CD163+ cells across all glomeruli was 70 (4-658), and the median maximum number of CD163+ cells within a single glomerulus was 20 (4-60); per-biopsy glomerular sampling adequacy and CD163^+^ cell counts are detailed in online supplemental table 5.

**Table 2:** Correlations of glomerular CD163^+^ cell counts (total and maximum per glomerulus) with clinical, serological and biomarker parameters in active lupus nephritis biopsies (n=15). Correlation coefficients are Spearman ρ; two-tailed p-values.

Variable	Total ρ	P	Max ρ	P	Direction
Proteinuria (g/g)	0.69	0.003	0.61	0.010	↑
Serum CD163 (ng/mL)	0.54	0.020	0.60	0.010	↑
Urinary CD163 (ng/mg creatinine)	0.65	0.006	0.63	0.009	↑
Renal biopsy NIH activity index	0.68	0.005	0.59	0.020	↑
Serum albumin (g/dL)	−0.40	0.050	−0.69	0.003	↓
C3 (mg/dL)	−0.50	0.051	−0.53	0.030	↓

**Figure 4:**
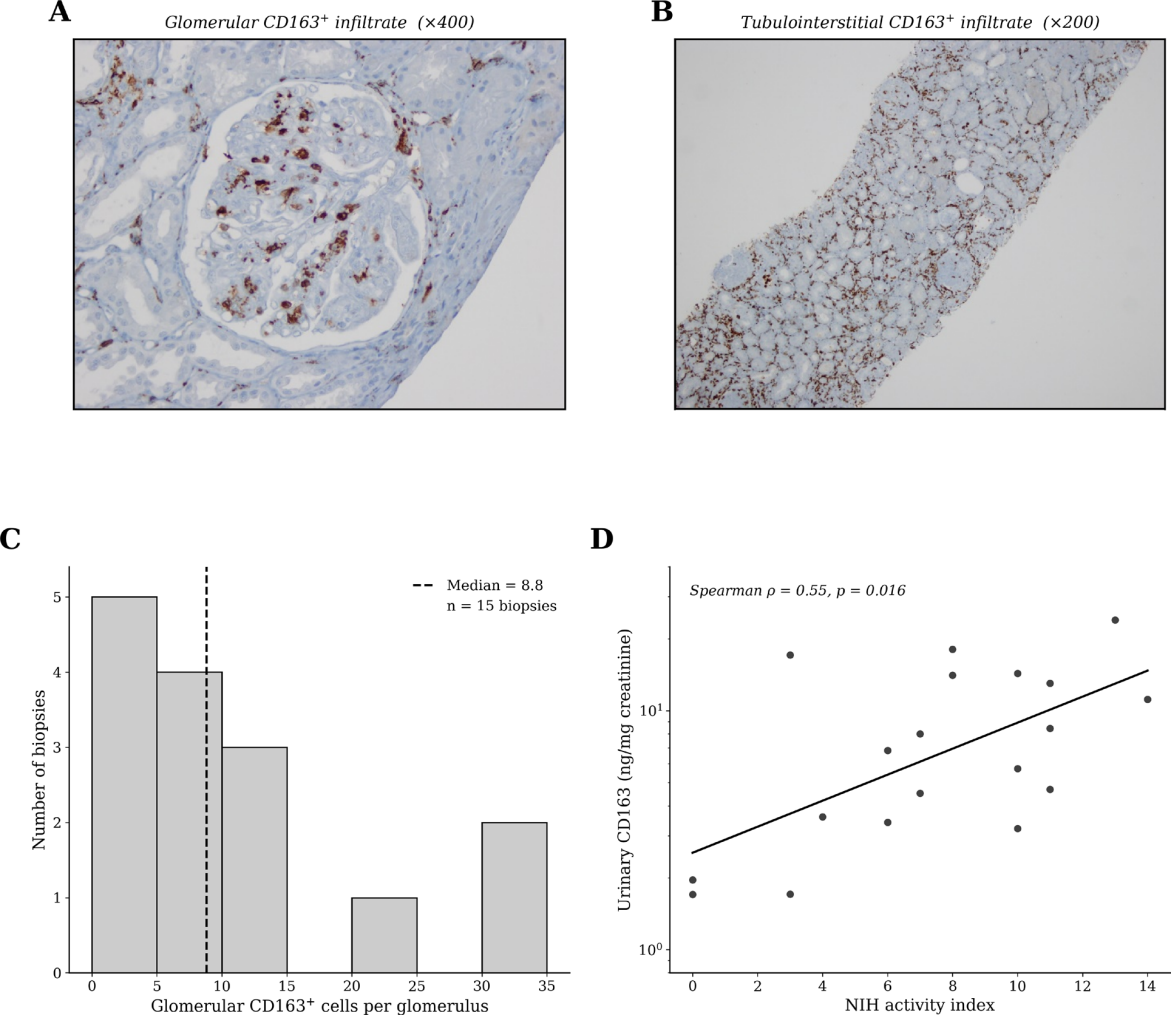
CD163 immunohistochemistry and its relationship with renal histological activity in active lupus nephritis. (**A**) Glomerular CD163^+^ macrophage infiltrate, localisation predominantly to mesangial areas and capillary lumens (original magnification ×400). (**B**) Tubulointerstitial CD163^+^ macrophage infiltrate (×200). CD163^+^ cells show brown (DAB) cytoplasmic staining; sections were counterstained with haematoxylin. (**C**) Distribution of glomerular CD163^+^ cell density across active LN biopsies (n=15); dashed line indicates the median. (**D**) Urinary CD163 (ng/mg creatinine) versus the NIH activity index analysed as a continuous variable in active LN biopsies (n=19); solid line, linear fit (Spearman ρ=0.55, p=0.016).

The number of glomeruli sampled per biopsy was a median of 10 (IQR 7–13.5; range 3–26; n=15 biopsies evaluable for glomerular CD163+ quantification). Median glomerular CD163+ cell density was 8.82 (IQR 3.75–13.38) cells per glomerulus (figure 4C). uCD163 correlated with the NIH activity index analysed as a continuous variable (ρ=0.55, p=0.016), but not with the chronicity index (ρ=0.09, p=0.72), consistent with specificity for active rather than fibrotic intrarenal processes (figure 4D). Among the three pure membranous (Class V) patients, uCD163 was below the positivity threshold in two (1.70 and 1.96 ng/mg) and elevated in one (13.84 ng/mg), whereas proliferative cases showed consistently higher values (median 8.00 ng/mg, IQR 4.52–14.08).

### Correlation of serum and urine soluble CD163 with clinical and laboratory parameters associated with disease activity in patients with active LN

sCD163 levels showed a significant positive correlation with clinical SLEDAI scores (ρ=0.43, p=0.01) and proteinuria (ρ=0.50, p<0.001). Conversely, sCD163 levels exhibited significant negative correlations with serum albumin (ρ=−0.53, p=0.002), C3 (ρ=−0.41, p=0.01) and C4 levels (ρ=−0.48, p=0.005).

uCD163 levels correlated positively with proteinuria (ρ=0.71, p<0.001) and cSLEDAI (ρ=0.60, p<0.001) across the SLE cohort. A detailed overview of the correlations between soluble CD163 levels and clinical/laboratory parameters is presented in online supplemental table 1.

### Correlation of histopathological parameters with clinical and laboratory parameters in patients with active LN

The total number of CD163+ macrophages in glomeruli correlated significantly with proteinuria (UPCR; ρ=0.69, p=0.003), the NIH activity index (ρ=0.68, p=0.005), urinary CD163 (ρ=0.65, p=0.006) and the sCD163 level (ρ=0.54, p=0.02) and negatively with serum albumin (ρ=−0.40, p=0.05) (table 2).

Notably, the maximum number of CD163+ cells within a single glomerulus exhibited significant positive correlations with proteinuria (ρ=0.61, p=0.01), sCD163 (ρ=0.60, p=0.01) and uCD163 (ρ=0.63, p=0.009). Furthermore, it correlated positively with the renal biopsy activity score (ρ=0.59, p=0.02) and negatively with serum albumin (ρ=−0.69, p=0.003) and C3 levels (ρ=−0.53, p=0.03). Detailed correlation data are provided in table 2.

### Diagnostic value of biomarkers in assessing disease activity

The ability of serum and urinary CD163 to discriminate between disease states was evaluated using ROC curve analysis, as detailed below.

#### Differentiation between active LN and extra-renal active SLE

ROC analysis was performed to evaluate the ability of CD163 levels to discriminate active LN from active extra-renal SLE. sCD163 showed no significant predictive value (AUC=0.53, 95% CI 0.33 to 0.71). In contrast, uCD163 demonstrated strong diagnostic potential with an AUC of 0.89 (95% CI 0.77 to 0.98).

A cut-off value of 4.52 ng/mg for uCD163 yielded a sensitivity of 70% and a specificity of 100%. The optimal cut-off for sCD163 (42.7 ng/mL) provided lower diagnostic performance (sensitivity 20%, specificity 100%). These findings indicate that uCD163, unlike sCD163, distinguishes patients with active LN from those with active extra-renal SLE (online supplemental figure 1B).

#### Differentiation between active LN and inactive LN

In distinguishing active LN from inactive LN, uCD163 demonstrated an AUC of 0.97 (95% CI 0.90 to 1.00). A cut-off value of 3.21 ng/mg for uCD163 provided a sensitivity of 85% and a specificity of 100%.

In contrast, sCD163 did not differentiate these groups (AUC=0.62, 95% CI 0.39 to 0.85); a cut-off of 16.6 ng/mL yielded a sensitivity of 65% and a specificity of 70%. These results confirm that uCD163 differentiates active LN from inactive LN (online supplemental figure 1A).

#### Differentiation between active LN and healthy controls

uCD163 also robustly distinguished active LN from healthy controls, with an AUC of 0.96 (95% CI 0.90 to 1.00). A cut-off of 3.21 ng/mg yielded a sensitivity of 85% and a specificity of 100% (online supplemental figure 1C). Diagnostic performance metrics for uCD163 across all three comparisons are summarised in online supplemental table 6.

#### Head-to-head comparison of uCD163 with conventional markers

To contextualise the diagnostic value of CD163 relative to conventional markers, we compared the discrimination of active versus inactive LN across six markers using ROC analysis with DeLong 95% CIs and pairwise DeLong tests (table 1). uCD163 (AUC 0.97) significantly outperformed serum CD163 (0.62; p=0.006) and anti-dsDNA positivity at sampling (0.73; p=0.002) and was statistically comparable to serum C3 (0.86; p=0.10), C4 (0.85; p=0.11) and proteinuria (p=0.25). UPCR showed perfect discrimination (AUC 1.00), as expected because group assignment was anchored on proteinuria; this comparison therefore does not credit uCD163 with diagnostic value incremental to proteinuria. Rather, the distinct contribution of uCD163 lies in its correlation with histological activity (NIH activity index and glomerular CD163+ density), which proteinuria does not capture.

## Discussion

LN is one of the leading causes of mortality and morbidity in SLE.^24^ Although conventional markers such as serum complement levels, anti-dsDNA antibodies and proteinuria are routinely used to monitor LN activity, they do not always parallel histopathological changes. Discordance between clinical and histological activity poses significant management challenges: clinically active patients may harbour quiescent histology, risking unnecessary immunosuppression, while those in apparent remission may still have subclinical renal inflammation driving progressive damage. Since repeat renal biopsy is impractical in routine care, reliable non-invasive biomarkers are needed and CD163 has emerged as a promising candidate.

Although uCD163 is considered a marker of LN activity, evidence directly linking circulating and urinary levels with renal tissue expression remains limited. We addressed this gap by establishing a triple correlation between serum, urine and intrarenal CD163 within the same cohort. Soluble CD163 levels were closely associated with CD163+ macrophage accumulation in renal biopsies and with longitudinal treatment response, providing histopathological support for the biological relevance of CD163 as a non-invasive indicator of renal inflammatory activity particularly in settings where repeat biopsy is not feasible.^25–28^

A recent single-centre cohort of 129 LN patients reported that urinary CD163 discriminated active from inactive LN with near-perfect accuracy and that its longitudinal change tracked treatment response and predicted impending flares.^29 30^ Our findings, complemented by paired same-day CD163 immunohistochemistry on every active LN biopsy, are concordant in direction and magnitude. Inclusion of an active extra-renal SLE comparator group, not addressed in earlier work, allowed us to evaluate whether the urinary signal reflects intrarenal macrophage activity specifically rather than systemic SLE activity. The strong correlation between uCD163 and intrarenal glomerular CD163+ macrophage density (ρ=0.65) lends histopathological support to the use of uCD163 as a non-invasive index of renal inflammation. Although glomerular CD163+ macrophage density correlated with both proteinuria and the NIH activity index, the two themselves showed only a positive, non-significant correlation (ρ=0.48, p=0.072), capturing overlapping but distinct aspects of disease; the lack of significance in this small cohort likely reflects limited power rather than true independence. Intraglomerular CD163+ macrophage accumulation may represent a shared substrate linking the two.

Prior studies have consistently demonstrated elevated sCD163 and uCD163 levels in active LN compared with inactive LN and healthy controls.^13 14 31 32^ Notably, uCD163 correlates with the histological activity index in LN biopsies^32^ and reflects treatment response at 12-month follow-up.^14^ Biopsy-based studies have similarly confirmed the predominance of CD163+ macrophages in active LN lesions and their association with chronicity indices.^15 27 33^

Importantly, uCD163 also distinguished active LN from active extra-renal SLE,^13 31^ a comparison not consistently addressed in previous studies.

CD163+ macrophage infiltration was prominently observed in active LN biopsies. The density of intrarenal CD163+ macrophages correlated with the biopsy activity index and with clinical markers of disease severity including proteinuria and hypocomplementaemia. These histopathological correlations provide tissue-level validation for soluble CD163 as a marker of active renal inflammation. Our cohort was predominantly proliferative (17/20, 85%), with only three pure membranous cases. As CD163 is shed from infiltrating M2c macrophages—most abundant in proliferative lesions—the urinary signal is likely driven mainly by proliferative disease, and uCD163 was below the positivity threshold in two of three membranous cases. The single membranous patient with markedly elevated uCD163 may reflect intrarenal macrophage activity beyond histological class; however, this should be interpreted cautiously given the small membranous subgroup, which precluded a class-stratified comparison. Pooled estimates should therefore be interpreted as reflecting predominantly proliferative LN, and dedicated evaluation in membranous LN is warranted.

When evaluating whether biomarker levels parallel remission in patients with active LN, we found that CD163 levels significantly decreased in serum and urine samples collected at the sixth month of treatment in patients who achieved remission.

The primary limitation is the relatively small sample size, which may affect generalisability. However, the study was conducted at a dedicated tertiary SLE/APS centre, with a consistent multidisciplinary team ensuring standardised clinical and histopathological assessments, thereby strengthening internal validity. The absence of a 6-month rebiopsy also precluded direct histopathological comparison with follow-up biomarker levels. We did not quantify total glomerular area, using the number of glomeruli sampled for normalisation instead. Prospective validation against complete renal response, time to flare and long-term eGFR preservation in larger multicentre cohorts will further define the role of uCD163 in clinical practice.

Although glucocorticoid doses differed across groups (online supplemental table 2), urinary CD163 did not correlate significantly with the corticosteroid dose at sampling (ρ=0.41, p=0.074). Rather, its renal specificity was anchored at the tissue level: uCD163 correlated with glomerular CD163+ macrophage density and the NIH activity index (table 2)—intrarenal measures that are not explained by systemic glucocorticoid exposure alone.

## Conclusion

Soluble uCD163 differentiates active from inactive LN and active extra-renal SLE and tracks treatment response. Both sCD163 and, more prominently, uCD163 correlated with intrarenal CD163+ macrophage density, supporting a link between non-invasive measurements and renal inflammation.

By integrating serum, urine, and renal tissue CD163 within the same cohort, this study links non-invasive measurements to intrarenal CD163+ macrophage density, reinforcing the plausibility of uCD163 as a marker of macrophage-driven renal inflammation. These findings support uCD163 as a clinically meaningful, non-invasive adjunct for monitoring LN activity, pending validation in larger multicentre cohorts.

## Supplementary material

10.1136/lupus-2026-002166 online supplemental figure 1

10.1136/lupus-2026-002166 online supplemental table 1

## Data Availability

Data are available upon reasonable request.
