# Supplementary material for: CD163: a potential biomarker for lupus nephritis activity
Source: Lupus Sci Med. 2026 Aug 10;13(2):e002166. doi: 10.1136/lupus-2026-002166 (PMC13475101; doi:10.1136/lupus-2026-002166)
Supplement: Supplementary file 1 [file lupus-13-2-s002.docx]

**Supplementary Material**

*This section contains supplementary tables (S1–S6) and one supplementary figure (S1) supporting the main results. Items are referenced throughout the main text by their respective numbers.*

**Supplementary Table S1.** *Correlations of serum and urinary CD163 (creatinine-normalized) with clinical and laboratory parameters in patients with active lupus nephritis (n = 20).*

| **Variable** | **sCD163 ρ** | **p** | **uCD163 ρ** | **p** | **qFDR (uCD163)** |
| --- | --- | --- | --- | --- | --- |
| Age (years) | 0.32 | 0.160 | 0.57 | **0.009** | **0.018** |
| cSLEDAI-2K | 0.43 | **0.010** | 0.60 | **0.001** | **0.003** |
| Proteinuria (g/g) | 0.50 | **0.001** | 0.71 | **0.001** | **0.003** |
| Serum albumin (g/dL) | −0.53 | **0.002** | −0.48 | **0.034** | **0.041** |
| C3 (mg/dL) | −0.41 | **0.010** | −0.51 | **0.021** | **0.032** |
| C4 (mg/dL) | −0.48 | **0.005** | −0.40 | 0.080 | 0.080 |

*Spearman correlation coefficients (ρ); two-tailed p-values. qFDR, Benjamini–Hochberg false discovery rate-adjusted q-value applied within the uCD163 correlation panel. Bold values indicate p < 0.05. uCD163 is creatinine-normalized (ng/mg).*

**Supplementary Table S2.** *Baseline demographics, clinical, and laboratory features of patients with SLE.*

| **Characteristic** | **Active LN (n=20)** | **Inactive LN (n=10)** | **Extra-renal active SLE (n=15)** | **p*** |
| --- | --- | --- | --- | --- |
| Female sex, n (%) | 16 (80) | 7 (70) | 14 (93.3) | 0.171 |
| Age (years), median (IQR) | 35 (19–55) | 33 (20–49) | 36 (18–68) | 0.690 |
| Disease duration (months), median (IQR) | 35.5 (6–180) | 69 (6–169) | 76 (0–436) | 0.002 |
| ***Immunosuppressive agentsᵃ, n (%)*** | | | | |
| Hydroxychloroquine | 11 (55) | 7 (70) | 11 (73.3) | 0.315 |
| Azathioprine | 2 (10) | 2 (20) | 3 (20) | 0.698 |
| Mycophenolate mofetil | 7 (35) | 7 (70) | 6 (40) | 0.184 |
| Glucocorticoids | 18 (90) | 8 (80) | 12 (80) | 0.388 |
| Dose of glucocorticoids (mg/day MP), median (IQR) | 28 (4–40) | 2 (1–4) | 8 (1–40) | 0.002 |
| Rituximabᵇ | 0 | 0 | 3 (20) | N/A |
| cSLEDAI-2K, mean ± SD | 13.2 ± 3.86 | 1 ± 1.05 | 9.46 ± 5.57 | <0.001 |
| SLICC/ACR damage score, median (IQR) | 0 (0–2) | 0.2 (0–2) | 0.7 (0–4) | N/A |
| Serum creatinine (mg/dL), median (IQR) | 0.72 (0.62–1.43) | 0.65 (0.55–0.76) | 0.7 (0.6–0.8) | 0.679 |
| eGFR (mL/min/1.73 m²), median (IQR) | 119.5 (43.7–126.6) | 115.5 (91.5–133.6) | 118.3 (96.2–125.4) | 0.789 |
| Serum C3 (mg/dL), mean ± SD | 65.2 ± 31.8 | 89 ± 31.9 | 103.8 ± 21.3 | <0.001 |
| Serum C4 (mg/dL), mean ± SD | 9 ± 5.2 | 18 ± 6.3 | 19 ± 8.2 | <0.001 |

**Three-group comparison; ᵃAt the time of enrolment; ᵇIn the last six months.*

*Abbreviations: ACR, American College of Rheumatology; cSLEDAI-2K, clinical Systemic Lupus Erythematosus Disease Activity Index 2000; eGFR, estimated glomerular filtration rate; IQR, interquartile range; LN, lupus nephritis; MP, methylprednisolone; SD, standard deviation; SLE, systemic lupus erythematosus; SLICC, Systemic Lupus International Collaborating Clinics.*

**Supplementary Table S3.** *Baseline demographic, clinical, laboratory, and histopathological features of patients with active and inactive lupus nephritis.*

| **Characteristic** | **Active LN (n=20)** | **Inactive LN (n=10)** |
| --- | --- | --- |
| Disease duration (months), median (IQR) | 35.5 (6–180) | 69 (6–169) |
| Duration of nephritis (months), median (IQR) | 0.5 (0–115) | 69 (6–169) |
| ***Histopathology*** | | |
| Class I, n (%) | 0 | 0 |
| Class II, n (%) | 0 | 1 (10) |
| Class III, n (%) | 4 (20) | 4 (40) |
| Class IV, n (%) | 13 (65) | 4 (40) |
| Class V, n (%) | 3 (15) | 1 (10) |
| Proliferative LNᵃ, n (%) | 17 (85) | 8 (80) |
| Non-proliferative LNᵇ, n (%) | 3 (15) | 2 (20) |
| NIH activity score, mean ± SD | 7.47 ± 4.07 | 7.80 ± 5.94 |
| NIH chronicity score, mean ± SD | 0.95 ± 1.22 | 0.80 ± 0.92 |
| ***Clinical and laboratory features*** | | |
| cSLEDAI-2K, mean ± SD | 13.2 ± 3.86 | 1 ± 1.05 |
| Haematuria, n (%) | 18 (90) | 0 |
| Proteinuria, n (%) | 20 (100) | 0 |
| Pyuria, n (%) | 7 (35) | 0 |
| Serum creatinine (mg/dL), median (IQR) | 0.72 (0.62–1.43) | 0.65 (0.55–0.76) |
| eGFR (mL/min/1.73 m²), median (IQR) | 119.5 (43.7–126.6) | 115 (91.5–133.6) |
| Serum albumin (g/dL), mean ± SD | 3 ± 0.8 | 3.7 ± 0.6 |
| Proteinuria (g/g), median (IQR) | 2.2 (1.31–4.79) | 0.04 (0–0.2) |
| Serum C3 (mg/dL), mean ± SD | 65.2 ± 31.8 | 89 ± 31.9 |
| Serum C4 (mg/dL), median (IQR) | 9 (5–22) | 18 (5–21) |
| Anti-dsDNA positivity, n (%) | 16 (80) | 0 |

*ᵃClass III ± V or Class IV ± V; ᵇPure Class I, II, or V.*

*Abbreviations: cSLEDAI-2K, clinical Systemic Lupus Erythematosus Disease Activity Index 2000; dsDNA, double-stranded DNA; eGFR, estimated glomerular filtration rate; IQR, interquartile range; LN, lupus nephritis; NIH, National Institutes of Health; SD, standard deviation.*

**Supplementary Table S4.** *Urinary CD163 positivity rates across study groups, using a cut-off defined as the mean + 2 SD of the healthy control group (2.32 ng/mg).*

| **Group** | **Positive / Total** | **Positivity rate (%)** | **p vs Active LN** |
| --- | --- | --- | --- |
| Active LN (n=20) | 17 / 20 | 85 | — |
| Inactive LN (n=10) | 1 / 10 | 10 | **<0.001** |
| Active extra-renal SLE (n=15) | 7 / 15 | 47 | **0.011** |
| Healthy controls (n=20) | 1 / 20 | 5 | **<0.001** |

*Positivity threshold = mean + 2 SD of healthy controls (uCD163 = 2.32 ng/mg). P values from Fisher's exact test versus active LN. Bold values indicate p < 0.05.*

**Supplementary Table S5.** *Per-biopsy glomerular sampling adequacy and CD163⁺ cell counts in active lupus nephritis biopsies evaluable for glomerular quantification (n = 15).*

| **Patient ID** | **LN class** | **Glomeruli sampled** | **Total CD163⁺ cells** | **Max per glomerulus** | **Density** |
| --- | --- | --- | --- | --- | --- |
| LN-01 | IV | 10 | 141 | 23 | 14.1 |
| LN-02 | IV | 4 | 35 | 15 | 8.8 |
| LN-03 | III | 13 | 13 | 3 | 1.0 |
| LN-04 | IV | 11 | 380 | 60 | 34.5 |
| LN-05 | V | 7 | 24 | 4 | 3.4 |
| LN-06 | IV+V | 9 | 108 | 18 | 12.0 |
| LN-07 | III | 12 | 73 | 12 | 6.1 |
| LN-08 | IV | 8 | 194 | 32 | 24.3 |
| LN-09 | III+V | 15 | 52 | 9 | 3.5 |
| LN-10 | IV | 10 | 162 | 28 | 16.2 |
| LN-11 | V | 6 | 16 | 6 | 2.7 |
| LN-12 | IV | 26 | 658 | 60 | 25.3 |
| LN-13 | III | 7 | 32 | 7 | 4.6 |
| LN-14 | IV+V | 13 | 112 | 14 | 8.6 |
| LN-15 | IV | 3 | 70 | 20 | 23.3 |

*Density = total CD163⁺ cells / glomeruli sampled (cells per glomerulus). All biopsies had ≥ 3 evaluable glomeruli; median 10 (IQR 7–13.5). Patient identifiers anonymized.*

**Supplementary Table S6.** *Diagnostic performance of urinary CD163 (creatinine-normalized) for discriminating active lupus nephritis from comparator groups.*

| **Comparison** | **AUC** | **95% CI** | **Cut-off (ng/mg)** | **Sensitivity (%)** | **Specificity (%)** |
| --- | --- | --- | --- | --- | --- |
| Active LN vs Inactive LN | **0.97** | 0.90–1.00 | 3.21 | 85 | 100 |
| Active LN vs Active extra-renal SLE | **0.89** | 0.77–0.98 | 4.52 | 70 | 100 |
| Active LN vs Healthy controls | **0.96** | 0.90–1.00 | 3.21 | 85 | 100 |

*AUC, area under the receiver operating characteristic curve; CI, confidence interval; LN, lupus nephritis; SLE, systemic lupus erythematosus. Urinary CD163 was normalized to spot urine creatinine and is expressed as ng of CD163 per mg of creatinine. 95% confidence intervals were derived from 1,000 bootstrap resamples. Optimal cut-off values were determined using the Youden index. Bold AUC values indicate AUC ≥ 0.85.*

**Supplementary Figure S1.** *Receiver operating characteristic curves of urinary CD163 (creatinine-normalised) for discriminating active lupus nephritis from comparator groups. (A) Active LN versus inactive LN. (B) Active LN versus active extra-renal SLE. (C) Active LN versus healthy controls. Open circles mark the Youden-optimal cut-off in each panel, with corresponding sensitivity and specificity. AUC values with 1,000-bootstrap 95% confidence intervals are shown.*
